# Effective Diagnosis and Treatment through Content-Based Medical Image Retrieval (CBMIR) by Using Artificial Intelligence

**DOI:** 10.3390/jcm8040462

**Published:** 2019-04-05

**Authors:** Muhammad Owais, Muhammad Arsalan, Jiho Choi, Kang Ryoung Park

**Affiliations:** Division of Electronics and Electrical Engineering, Dongguk University, 30 Pildong-ro 1-gil, Jung-gu, Seoul 04620, Korea; malikowais266@gmail.com (M.O.); arsal@dongguk.edu (M.A.); choijh1027@dongguk.edu (J.C.)

**Keywords:** medical treatment, content-based medical image retrieval (CBMIR), artificial intelligence, residual network (ResNet), medical image classification

## Abstract

Medical-image-based diagnosis is a tedious task‚ and small lesions in various medical images can be overlooked by medical experts due to the limited attention span of the human visual system, which can adversely affect medical treatment. However, this problem can be resolved by exploring similar cases in the previous medical database through an efficient content-based medical image retrieval (CBMIR) system. In the past few years, heterogeneous medical imaging databases have been growing rapidly with the advent of different types of medical imaging modalities. Recently, a medical doctor usually refers to various types of imaging modalities all together such as computed tomography (CT), magnetic resonance imaging (MRI), X-ray, and ultrasound, etc of various organs in order for the diagnosis and treatment of specific disease. Accurate classification and retrieval of multimodal medical imaging data is the key challenge for the CBMIR system. Most previous attempts use handcrafted features for medical image classification and retrieval, which show low performance for a massive collection of multimodal databases. Although there are a few previous studies on the use of deep features for classification, the number of classes is very small. To solve this problem, we propose the classification-based retrieval system of the multimodal medical images from various types of imaging modalities by using the technique of artificial intelligence, named as an enhanced residual network (ResNet). Experimental results with 12 databases including 50 classes demonstrate that the accuracy and *F1.score* by our method are respectively 81.51% and 82.42% which are higher than those by the previous method of CBMIR (the accuracy of 69.71% and *F1.score* of 69.63%).

## 1. Introduction

Over the past few decades, computer-aided diagnosis (CAD) tools and techniques have been widely adopted for better medical treatment [1,2]. These modern tools support medical experts in many areas such as medical diagnosis and treatment for any specific disease or injury. In the current era of medical science, many computer-aided tools provide visual information for diagnosis and treatment such as magnetic resonance imaging (MRI), X-ray, angiography, computed tomography (CT), digital mammography, optical projection tomography (OPT), colonoscopy, ultrasonography, optical endoscopy, nuclear medical imaging‚ and positron-emission tomography (PET) [3,4]. These various medical imaging modalities provide visual insight into different hidden body organs, thus enabling better diagnosis and treatment. Medical image analysis is a challenging task due to the complex structure of body organs‚ and medical experts are required for accurate interpretation [4]. To arrive at a sound decision about a serious medical condition, past relevant cases are explored by many medical experts. This practice can facilitate better diagnosis and treatment. However, due to the enormous number of medical visual records generated by many different imaging modalities, it is very difficult and time-consuming to retrieve relevant cases. This problem can be resolved by using a computer-based medical image retrieval system (MIRS), which helps medical experts in retrieving past relevant cases from previous patients’ databases.

To develop such a MIRS, image classification is the key challenge due to the existence of highly correlated visual features among different classes, which ultimately results in low retrieval performance. This problem can be solved by using advanced machine learning tools and techniques that may result in better classification performance. Better performance can be achieved by exploring hidden features that the human visual system (HVS) finds very difficult to identify. In the past few years, significant advances have been made in the area of machine learning and artificial intelligence (AI) including deep learning framework [5]. The key idea behind deep learning is analogous to the operation of human brain, in which information is also processed through multiple layers of transformation [5,6].

Deep learning methods have shown significant performance in general content-based image retrieval (CBIR) applications [7]. In the past few years, deep learning models have made significant contributions in various medical domains [8,9] including brain tumor detection [1], blood flow quantification and visualization, diabetic retinopathy (DR), and many cancer detection applications. However, these methods are still in the developmental phase for content-based medical image retrieval (CBMIR) tasks, due to the rapid growth in medical imaging technology [10]. This paper mainly focuses on the analysis of different deep learning models used in medical image classification and retrieval. We analyze in depth the performance of the most recent convolutional neural network (CNN) models from the following standpoints, by considering: (1) different configuration modes of CNN models, (2) feature selection from different layers within a network, (3) training from scratch and fine-tuning, and (4) modification of the pre-trained model. In this way, we proposed the best CNN model after modifying the existing model to obtain the best classification accuracy. Finally, we provide a first pre-trained model for a heterogeneous medical database including the number of classes captured by different modalities, which is our main contribution, and we have also made our pre-trained model and image indices of experimental images publicly available for other researchers.

CBMIR is an active area of research with significant applications in routine clinical diagnostic aid, medical education, and research. Many solved cases related to different diseases can be stored in a picture archiving and communication system (PACS) or in CBMIR systems with comprehensive patient record and treatment details. In the future, similar cases can be diagnosed in less time by exploring such previous records. In this way, medical experts can save precious time and improve diagnosis and treatment. Moreover, CBMIR is also helpful in medical teaching and research areas.

The rest of the paper is organized as follows: In Section 2, we describe the related studies. Section 3 summarizes the main contribution of this paper. The proposed CNN-based classification method for medical image retrieval is described in Section 4. In Section 5 and Section 6, the experimental setup, performance analysis, and discussions are presented. Finally, Section 7 concludes our research.

## 2. Related Works

The present era of digital technology has made a significant contribution to medical science. The number of medical imaging modalities is growing rapidly with improvements in biomedical sensors and high-throughput image acquisition technologies. These devices generate an enormous collection of heterogeneous medical images that make a significant contribution to disease analysis and treatment. A medical expert can make a better diagnosis related to a similar situation in the past by retrieving relevant cases from this enormous collection of medical images. Before the advent of machine learning (ML) and AI algorithms, it was considered a tedious task to explore the huge multimodal database for getting assistance related to any complex problem. Hence, it is important to evolve an efficient MIRS that will support medical experts and thus improve diagnosis and treatment.

Conventional text-based image retrieval systems use certain textual tags that images are often manually annotated with as search keywords. Due to the enormous collection of heterogeneous medical image databases, this manual annotation task is very tedious and time-consuming. In many hospitals, the PACS [11] is deployed to manage a very large collection of medical images that is compatible with the digital imaging and communications in medicine (DICOM) file format [12]. This framework utilizes the textual information stored in the DICOM header for image retrieval; the header contains a patient identifier (ID), name, date, modality, body parts examined, etc. This header information is lost when a DICOM image is converted into another image format for efficient storage and communication such as tagged image file format (TIFF), joint photographic experts group (JPEG), portable network graphics (PNG), etc. To resolve this problem, CBMIR systems have been proposed by many researchers to assist medical experts. However, these systems are application-specific and can store or retrieve a specific type of medical image, e.g., a retrieval system for X-ray images of the chest as proposed in [13].

Although many researchers have studied the CBMIR by using handcrafted features [14,15,16,17,18,19,20,21,22,23,24,25,26], the overall performance of the existing systems is still low due to the growing heterogeneous medical images of multiclass database and conventional ML techniques. These techniques are unable to decrease the “semantic gap,” which is the information lost by converting an image (i.e., a high-level representation) into its visual features (i.e., a low-level representation) [27]. Recently, a significant breakthrough has occurred in the ML domain with the advent of the deep learning framework, which comprises many efficient ML algorithms that can show high-level abstractions in visual data with a minimum semantic gap [28]. Ultimately, these layers extract the complex deep features from the input data in a fully systematic way. Finally, the deep network learns from these features without using other handcrafted features.

In recent studies, a significant breakthrough in deep learning has been done in the medical domain, and they are classified into two categories of single modality-based [29,30,31,32,33,34,35,36] and multiple modalities-based methods [28] of imaging.

As the single modality-based method, a two-stage CBMIR framework is presented for automatic retrieval of radiographic images [29]. In the first stage, the main class label is assigned by using CNN-based features, and in the second stage, outlier images are filtered out from the predicted class on the basis of low-level edge histogram features. Another CNN-based system is presented in [30] for categorization of interstitial lung diseases (ILDs) patterns by extraction of ILD features from the selected dataset. In [31], a convolutional classification restricted Boltzmann machine (RBM)-based framework is proposed for analyzing the lung CT scan by combining both generative and discriminative representation learning. A CNN-based automatic classification of peri-fissural nodules (PFN) is presented in [32], which has high relevance in the context of lung cancer screening. In [33], a two-stage multi-instance deep learning framework is presented for the classification of different body organs. In the first stage, a CNN is trained on local patches to separate discriminative and non-informative patches from training data samples. The network is then fine-tuned on extracted discriminative patches for the classification task. A detailed analysis of deep learning in CAD is presented in [37]. Three main characteristics (i.e., different CNN architectures, dataset scale, and transfer learning) of CNN are explored in this work. A deep CNN model pre-trained on the general dataset is then fine-tuned for a large collection of multimodal medical image databases. A fully automatic 3D CNN framework to detect cerebral microbleeds (CMBs) from MRI is proposed in [34]. CMBs are small hemorrhages near blood vessels whose detection provides deep insight into many cerebrovascular diseases and cognitive dysfunctions. In [35], an efficient CNN training method is proposed by dynamically choosing negative samples (misclassified) during the training process, which shows better performance in hemorrhage detection within a color fundus image. A multiview convolutional network (ConvNets)-based CAD system is proposed [36] for detecting pulmonary nodules from lung CT scan images.

As the multiple modalities-based method, a deep-learning-based framework for multiclass CBMIR is recently proposed in [28] that can classify multimodal medical images. In this framework, an intermodal dataset that contains twenty-four classes with five modalities (CT, MRI, fundus camera, PET, and OPT) is used to train the network.

The maximum numbers of classes can usually increase the usability of CBMIR system in healthcare medical application [28]. In addition, it is reported that a large number of classes can help the medical expert in exploring the specific class of disease from a huge collection of medical record according to [38] and healthcare professional. Nevertheless, in previous researches, the maximum numbers of classes to be dealt with were limited as 31 [20,29], and we increased the numbers of classes as 50 in our research. For this purpose, we propose a deep-feature-based medical image classification and retrieval framework by using the enhanced residual network (ResNet) for CBMIR of large numbers of classes with nine modalities (CT, MRI, fundus camera, PET, OPT, X-ray, ultrasound, endoscopy, and visible light camera). The strengths and weaknesses of our proposed and existing methods are summarized in Table 1.

## 3. Contribution

Our research is novel in the following six ways compared to previous works. The brief definitions of the closed-world, open-world, and mixed-world configurations in the 1st contribution are as follows. The closed-world configuration means the case that the classes in training are same as those used in testing whereas the open-world configuration represents the case that the classes in training are different from those used in testing and the classes in testing are unknown. The mixed-world configuration is the combination of the closed-world and open-world configurations, and it means the case that some parts of testing data are not known in training process whereas the others of testing data are known in training process. Detail definitions of these three configurations are explained in Section 5.3.5.

- This is the first approach toward classifying the large collection of multiclass medical image databases with multiple modalities based on the deep residual network in the closed-world, open-world, and mixed-world configurations. Different from our research, most of the previous studies [10,28,29,30,31,32,33,34,35,36] have been conducted only in a closed-world configuration.
- In general, the problem for classification with larger numbers of classes is more difficult than that with fewer numbers of classes. Based on the theories in pattern recognition, the inter-distance between classes in case of larger numbers of classes becomes smaller than that in case of fewer numbers of classes. This increases the possibility of overlapping of data from different classes, and consequent classification error is increased [39,40,41]. It is also experimentally confirmed that the previous method [28] shows the accuracy of *F1.score* as 69.63% with 50 classes whereas it presents the accuracy of *F1.score* as 99.76% with 24 classes [28].
- In our proposed medical image classification and retrieval framework, we modified the conventional ResNet50 [42] CNN model by replacing its last 7 × 7 average pooling layer with a 7 × 7 × 2048 convolutional layer. Finally, the number of nodes in the last fully connected (FC) layer is also adjusted according to the number of classes in our dataset.
- We deeply analyze the characteristics of various CNNs for multiclass medical images, and then check how a specific CNN structure can influence the classification performance of multiclass medical images.
- We compare the performance of state-of-the-art CNN models, not only through fine-tuning and tuning from scratch but also against different handcrafted approaches. Our analysis is more detailed, in contrast to previous studies [10,28], which provided only a limited performance comparison for a small number of databases.
- We analyze the performance of a CNN model based on feature selection from the different layers of the network.
- We have made our trained model and image indices of experimental images publicly available through [43], so that other researchers can evaluate and compare its performance.

## 4. Proposed Method

### 4.1. An Overview of the Proposed Approach

**Figure 1:**
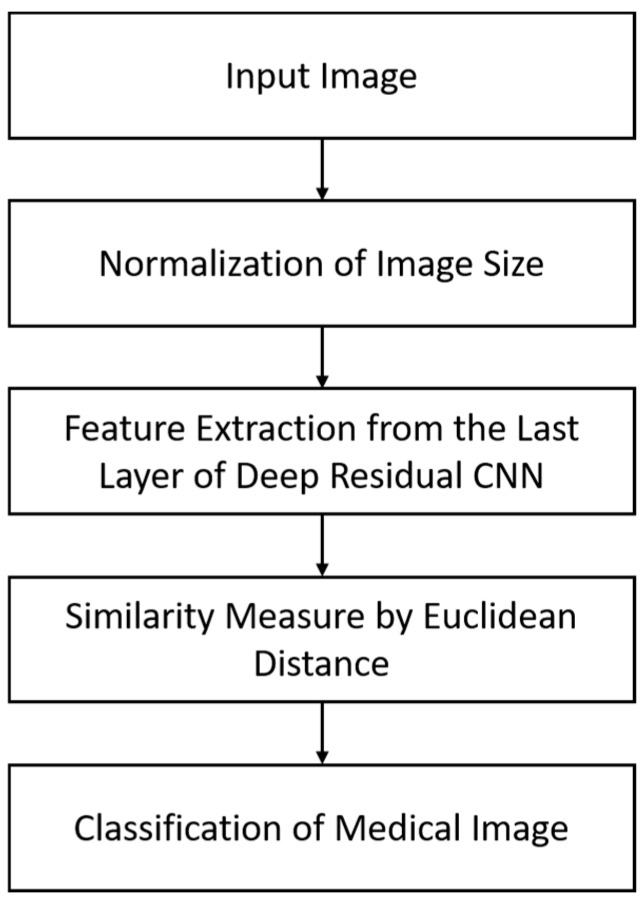
Overall procedure of the proposed method for classification.

Figure 1 presents a brief flowchart for the classification of medical images by using our modified deep residual CNN framework. In the first step, the given medical image was resized into 224×224×3 for input to our CNN model. The resized image was then passed to a deep residual CNN model for feature extraction from the last convolutional layer. In this way, a deep feature vector (1×2048) was obtained, which represents the complex hidden structure of the given input image (i.e., high-level representation) as a feature vector (i.e., low-level representation). This extracted feature vector was compared one by one with the labeled feature vectors in the database by measuring the Euclidean distance. Finally, a class label was assigned to the given input image on the basis of the minimum distance score. A detailed explanation of our proposed model is provided in subsequent sections.

### 4.2. The Structure of our Modified Deep Residual CNN

In our proposed medical image classification and retrieval framework, we modified the conventional ResNet50 [42] CNN model by replacing its last 7 × 7 average pooling layer with a 7 × 7 × 2048 convolutional layer. The reasons for using 7 × 7 × 2048 convolutional layer are as follows. Compared to the classification of general images, the classification of medical images has the problems of high inter-class similarity. Therefore, more features which can be useful for the classification should be extracted from the CNN. The original ResNet50 [42] obtains the feature map of 1 × 1 × 2048 from the previous feature map of 7 × 7 × 2048 by using average pooling layer including one filter of 7 × 7, which can cause the loss of useful features. To solve this problem, our revised ResNet50 obtained the feature map of 1 × 1 × 2048 from the previous feature map of 7 × 7 × 2048 by using the additional convolution layer (Conv6 in Table 2) including 2048 filters of 7 × 7 × 2048, which can reduce the loss of useful features. In addition, the filter coefficients of average pooling layer in original ResNet50 are fixed ones, whereas the optimal filter coefficients of the additional convolutional layer in our revised ResNet50 can be obtained by training. In order to prove this, we experimentally compared the accuracies by original ResNet50 [42] using the average pooling layer including 1 filter of 7 × 7 with those by our revised ResNet50 using the additional convolution layer including 2048 filters of 7 × 7 × 2048. The accuracies by our method are higher than those by original ResNet50 [42].

**Figure 2:**
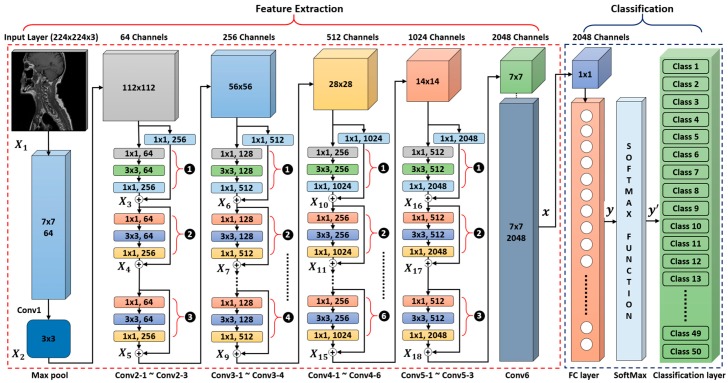
Overview of the proposed deep convolutional neural network (CNN) architecture used for feature extraction and classification.

Finally, the number of nodes in the last FC layer is adjusted according to the number of classes in our dataset. The modified structure of the CNN with the complete layer configuration is presented in Figure 2 and Table 2.

**Figure 3:**
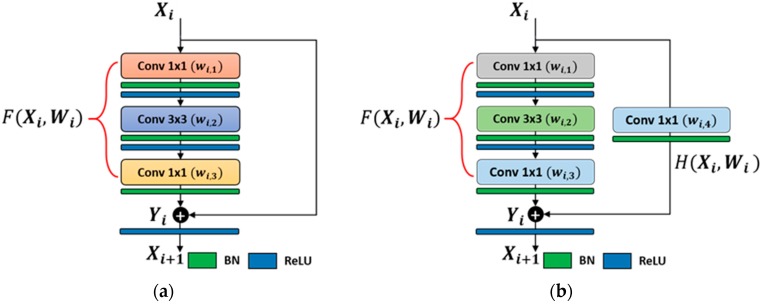
The residual building block of our modified ResNet50 with (**a**) identity-mapping-based residual unit, and (**b**) 1×1 convolutional-mapping-based residual unit.

Our modified deep residual CNN network was made up of multiple residual units that can be considered as a basic building block. These residual units included both identity-mapping-based and 1 × 1 convolutional-mapping-based shortcut connections [42]. The shortcut connection in identity-mapping-based residual unit mapped the input feature map as it was, without changing its size and depth. On the other hand, the shortcut connection in the 1 × 1 convolutional-mapping-based residual unit increased the depth of the input feature map. Our deep residual network contained a total of 16 residual units, in which there were 12 identity mapping units and four convolutional mapping units of 1 × 1, as shown in Figure 3. Using more residual units as identity mapping decreases the complexity and training time. Furthermore, both identity and 1 × 1 convolutional shortcut connections make information propagation smooth in both forward and backward directions [44].

The detailed layer configuration for our model is given in Table 2. Conv1–Conv6 was the convolutional layers stack in which Conv2–Conv5 represent the group of convolutional layers. Each individual group comprised multiple residual units including only one 1×1 convolutional-mapping-based residual unit and multiple identity-mapping-based residual units. The number of identity mapping units was different in each group, which is represented by the number of iterations in Table 2. In addition, max pool was a subsampling layer that was used to select the maximum value in a subregion of the feature map defined by the kernel size. Its main purpose was to reduce the feature map size by preserving information on key features. Finally, the FC layer, SoftMax, and the classification layer were used to classify the features extracted from the previous convolutional layers.

#### 4.2.1. Feature Extraction

The convolutional layer stack was used for feature extraction by applying a traditional 2D convolution operation using a different number of filters with different sizes. These filters contained learnable parameters that were determined during the training procedure. When convolution was applied, the output feature map size changed depending upon the filter size, number of filters, the stride values for the horizontal and vertical directions, and the range of filter movement using padding options. All these parameters, known as hyperparameters, were defined during the network construction phase. Therefore, they were very important for constructing an efficient model. In our deep residual CNN, Conv1 had 64 filters of 7×7×3 and it explored the given input image $X_{1}$ of 224×224×3, in both the horizontal and vertical directions with a stride of two pixel units, and a padding of three pixel units in both directions. The max pool layer had one filter of 3×3 pixels that explored the output feature map $X_{2}$ of Conv1 in both the horizontal and vertical directions with a stride of two pixel units for each input channel, and generated a down-sampled feature map of 56×56×64.

Conv2–conv5 was the group of multiple convolutional layers that comprise multiple residual units. In each group, there was only one convolutional mapping unit at the start, followed by multiple identity mapping units. As given in Table 2, in the first group (Conv2), Conv2–1 presents the first convolutional-mapping-based residual unit, consisting of four convolutional layers with filters $w_{2,1},w_{2,2},w_{2,3},w_{2,4}$. In this residual unit, the first three and fourth convolutional layers were connected in a parallel fashion as shown in Figure 3b. The first three layers performed the convolution operation in sequential order for a given input $X_{2}$ by applying filters $w_{2,1},w_{2,2},\;w_{2,3}$ and generating the intermediate feature map as $F\left(X_{2},W_{2}\right)$. The fourth layer converts the given input $X_{2}$ as $H\left(X_{2},W_{2}\right)$ by applying a 1×1 filter $w_{2,4}$ to equalize the depth size of $X_{2}$ according to $F\left(X_{2},W_{2}\right)$. Finally, the output feature map $X_{3}$ of 56×56×256 was obtained by adding $F\left(X_{2},W_{2}\right)$ and $H\left(X_{2},W_{2}\right)$. Conv2–2 presents the first identity-mapping-based residual unit including three convolutional layers, as shown in Figure 3a. These three layers further processed the output $X_{3}$ in sequential order by applying three different filters $w_{3,1},w_{3,2},w_{3,3}$ and generated the intermediate feature map $F\left(X_{3},W_{3}\right)$. The final feature map $X_{4}$ of 56×56×256 was generated by adding $F\left(X_{3},W_{3}\right)$ and the previous output feature map $X_{3}$. Similarly, Conv2–3 was the second identity-mapping-based residual unit, which performsed the same operation as in Conv2–2 and generated the feature map $X_{5}$ of 56×56×256.

Similarly, all the other convolutional-mapping-based and identity-mapping-based residual units in groups Conv3, Conv4, and Conv5 performed the same operation as in Conv2. The only difference was the different number of filters and identity-mapping-based residual units in each group. Due to the different number of filters in each group, the input feature map depth also increased. Furthermore, the input feature map size decreased by a factor of two after passing through each successive group. The reason is that in each group, the first convolutional-mapping-based residual unit considers a unit stride of two pixels. In conclusion, each group, Conv3, Conv4, and Conv5, generated output feature maps ($X_{9},\;X_{15}$, and $X_{18}$) of 28×28×512, 14×14×1024, and 7×7×2048, respectively, as shown in Table 2. Finally, the optimal feature vector x of 1×2048 was obtained after convolving the output $X_{18}$ of Conv5 with the last convolutional layer Conv6 using a filter of 7×7×2048. Batch normalization and the rectified linear unit (ReLU) activation function were also applied after each convolutional layer on the basis of the mean and standard deviation of the data. The final feature vector x was further used as the input to the FC layer.

#### 4.2.2. Classification

In our deep residual CNN method, we considered two classification architectures separately for both the training and testing phases. The 1st classification architecture used the 50 output nodes of classification layer in Table 2. For example, if the 2nd output node of classification layer with one input image showed the higher value than those from the other 49 nodes, the input image was determined as the class 2. The 1st classification architecture was used only for closed-world configuration.

The 2nd classification architecture determines the class of input image based on the Euclidean distance matching with the 2048 features extracted by Conv6 of Table 2. For example, if the distance between the 2048 features of input and 2048 mean feature vector of class 3 was the smallest, the input image was determined as class 3. The 2nd classification architecture was used for both open-world and mixed-world configurations.

**Figure 4:**
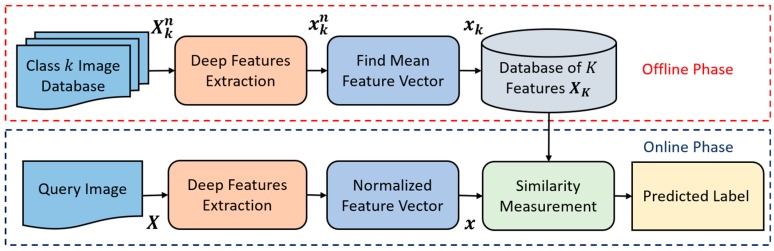
Proposed deep-feature-based variable node classification (VNC) framework.

For example, in open-world configuration, the data of *C*_1_, *C*_2_, … *C*_25_ were used for the training of CNN whereas those of *C*_26_, *C*_27_, … *C*_50_ were used for testing. Although the data of *C*_26_, *C*_27_, … *C*_50_ were not seen during the training, the 25 sets of 2048 mean feature vectors from *C*_26_, *C*_27_, … *C*_50_ were calculated and stored at our database in advance (the offline phase of Figure 4) for the Euclidean distance matching. For example, in mixed-world configuration, half of the data of *C*_1_, *C*_2_, … *C*_40_ were used for training of CNN whereas the other half data of *C*_1_, *C*_2_, … *C*_40_ and the whole data of *C*_41_, *C*_42_, … *C*_50_ were used for testing. In this case, the 10 sets of 2048 mean feature vectors from *C*_41_, *C*_42_, … *C*_50_ were calculated and stored at our database in advance (the offline phase of Figure 4) for the Euclidean distance matching.

The reason is that a variable number of testing classes can be considered in open-world and mixed-world configurations as compared to the closed-world configuration. In the closed-word configuration, the number of training and testing classes remained the same, and therefore there was no need to use a separate classifier for this operational mode. In this way, the FC part of our modified CNN model was used during the training phase. In the testing phase, our proposed deep-feature-based variable node classification (VNC) framework was deployed for class-prediction-based retrieval, whereas the feature extraction part remained the same in both the training and testing phases. In the training phase, a fully connected part mainly comprised the stack of the FC layer, the SoftMax layer, and the classification layer as shown in Figure 2. The FC layer (including the number of nodes which is equal to the total number of classes) was followed by the last convolutional layer Conv6 of the feature extraction part. The main purpose of this layer was to identify the larger patterns by combining all the features learned by the previous layers across the image. It multiplied the input feature vector obtained from Conv6 by a weight matrix and then added a bias vector. The next SoftMax layer of the classification part converted the output of the FC layer in terms of probability by applying the softmax function [45]. Finally, the classification layer took the output from the SoftMax layer and assigned each input to one of the 50 mutually exclusive classes using the cross-entropy function [45].

In the testing phase, the deep-feature-based VNC framework was implemented to classify the input query image in all configuration modes (i.e., closed-world, open-world, and mixed-world). A simple flow diagram of the proposed VNC framework was represented in Figure 4. There were two operational phases in the proposed VNC framework as in any general CBIR system, called the offline and online phases. In the offline phase, a database of K mean feature vectors was built from the available training dataset. For each individual class, a single mean feature vector was obtained. In this way, a set consisting of a total of 50 mean feature vectors was obtained from the entire training dataset. In our proposed work, the offline phase was mostly used in the open-world and mixed-world configurations in which the reference feature database can be updated for new classes without repeating the training process. In the online phase, the classification was performed for a given input query image by extracting and comparing its feature vector with the set of mean feature vectors by taking the $L_{2}$-norm. Ultimately, the final class label was assigned on the basis of the minimum distance. In this way, efficient class-prediction-based image retrieval was performed by retrieving the required images from the selected class label rather than by exploring the entire dataset.

## 5. Experimental Setup and Performance Analysis

Several experiments were performed to evaluate our deep-feature-based method from various perspectives. Different comparisons were made with many deep CNN and handcrafted feature-based methods. For better performance analysis in a real-world scenario, the settings of the experiments were made in three different configuration modes (i.e., closed-world, open-world, and mixed-world). In this section, we describe the details of the selected dataset, experimental configurations as well as observations, and analysis of the results.

### 5.1. Dataset and Experimental Protocol

In our research, we focused on the classification-based retrieval of medical image having multiple classes with multiple imaging modalities. Medical image computing and computer assisted intervention (MICCAI) grand challenges share the medical images [46], but most of these images were used for the purpose of detection and segmentation instead of classification-based retrieval. Therefore, we did not use the benchmark of MICCAI grand challenges, but we categorized 12 different publicly available databases [47,48,49,50,51,52,53,54,55,56,57,58] into 50 different classes (i.e., *C*1 to *C*50) on the basis of different medical imaging modalities, body organs, and disease types. Because our experimental images were not collected by us, we cannot make them open to other researchers. Instead, we made the websites [47,48,49,50,51,52,53,54,55,56,57,58] with the image indices of our experimental images and our trained CNN models available to other researchers through [43] in order for fair comparisons with our method.

**Figure 5:**
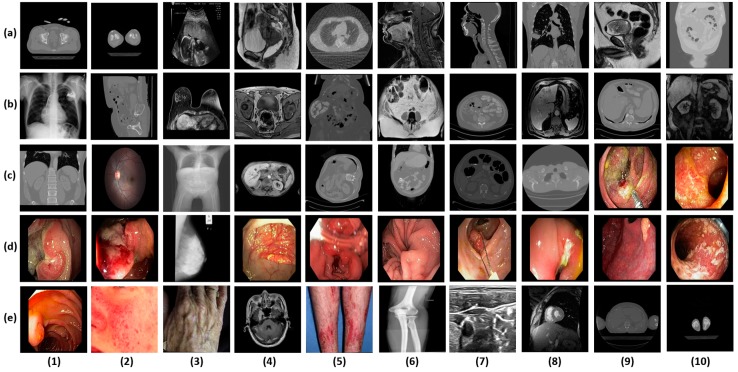
Examples from each class of total 50 classes. (**a-1**) Hip computed tomography (CT); (**a-2**) knee CT; (**a-3**) ultrasound; (**a-4**) cervix magnetic resonance imaging (MRI); (**a-5**) 4D lung CT; (**a-6**) head, neck MRI; (**a-7**) head, neck CT; (**a-8**) lung CT; (**a-9**) bladder MRI; (**a-10**) bladder CT; (**b-1**) chest X-Ray; (**b-2**) urography CT; (**b-3**) breast MRI; (**b-4**) prostate MRI; (**b-5**) uterus CT; (**b-6**) rectum MRI; (**b-7**) ovary CT; (**b-8**) liver MRI; (**b-9**) liver CT; (**b-10**) kidney, renal MRI; (**c-1**) kidney, renal CT; (**c-2**) retina; (**c-3**) CT topogram; (**c-4**) pancreas MRI; (**c-5**) pancreas CT; (**c-6**) stomach CT; (**c-7**) colonography CT; (**c-8**) esophagus CT; (**c-9**) malignant tumors; (**c-10**) sigmoid colon; (**d-1**) rectum; (**d-2**) colon; (**d-3**) breast mammogram; (**d-4**) caecum; (**d-5**) duodenal bulb; (**d-6**) normal esophagus; (**d-7**) benign tumors; (**d-8**) Crohn’s disease; (**d-9**) gastric fundus; (**d-10**) ulcerative colitis; (**e-1**) upper endoscopy; (**e-2**) facial acne; (**e-3**) hand, foot allergies; (**e-4**) brain MRI; (**e-5**) legs, arms allergies; (**e-6**) bones X-rays; (**e-7**) neck nerves; (**e-8**) cardiac MRI; (**e-9**) shoulder CT; (**e-10**) ankle CT.

**Figure 6:**
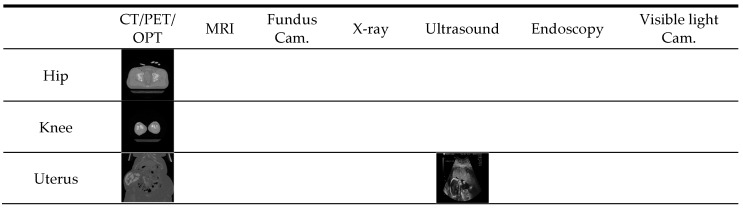

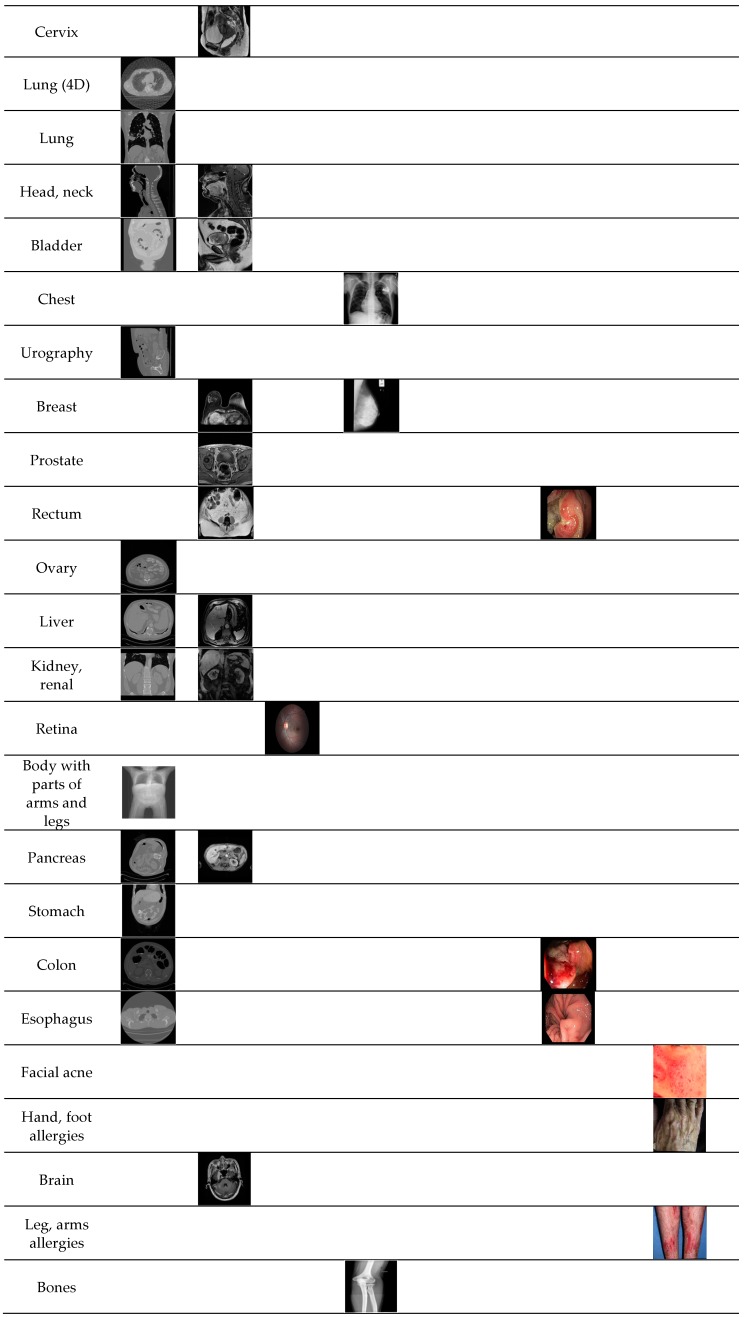

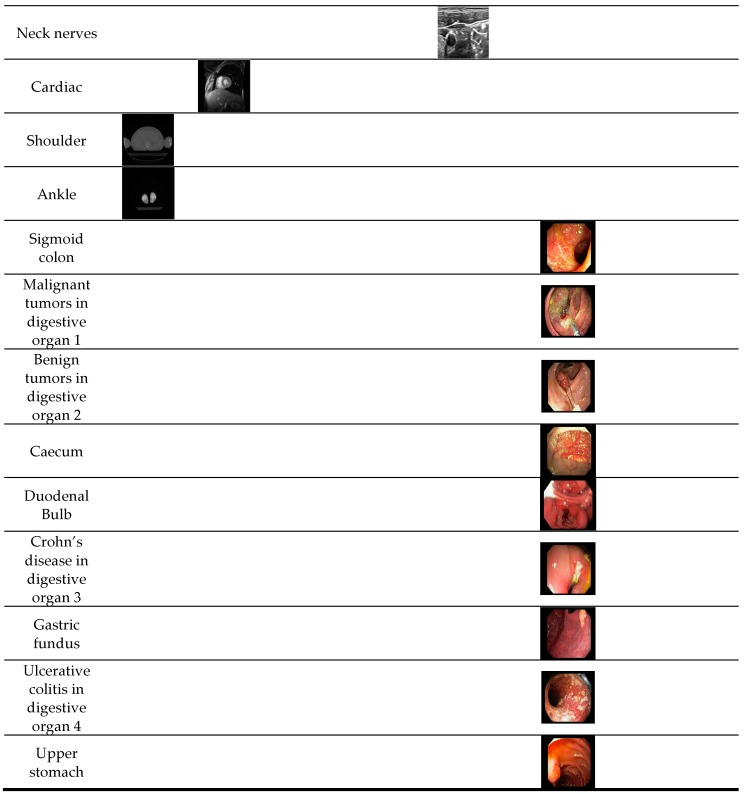
Examples of experimental data of Figure 5 according to the anatomical district (row) and imaging modality (column).

**Figure 7:**
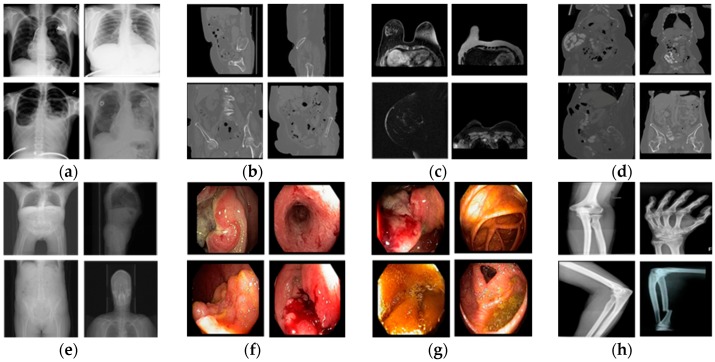
Selected example images for showing high intra-class variance; (**a**) chest X-ray in Figure 5b-1; (**b**) urography CT in Figure 5b-2; (**c**) breast MRI in Figure 5b-3; (**d**) uterus CT in Figure 5b-5; (**e**) CT topogram in Figure 5c-3; (**f**) rectum in Figure 5d-1; (**g**) colon in Figure 5d-2; (**h**) bones X-rays in Figure 5e-6.

**Figure 8:**
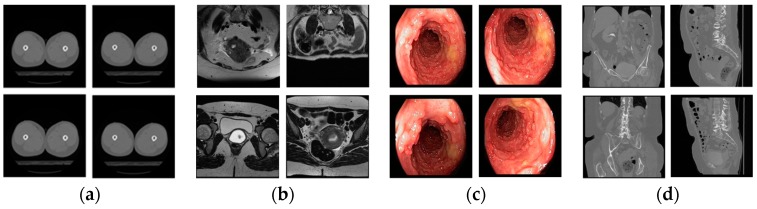

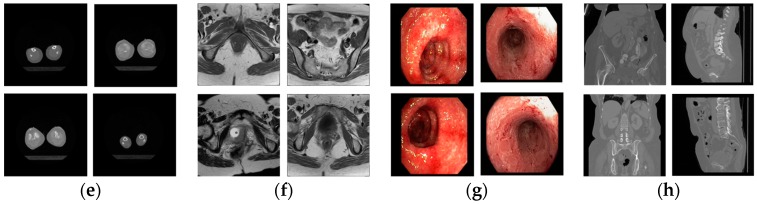
Selected example images for showing high inter-class similarity; (**a**,**e**) hip CT in Figure 5a-1 and knee CT in Figure 5a-2, respectively; (**b**,**f**) cervix MRI in Figure 5a-4 and bladder MRI in Figure 5a-9, respectively; (**c**,**g**) benign tumors in Figure 5d-7 and Rectum in Figure 5d-1, respectively; (**d**,**h**) urography CT in Figure 5b-2 and uterus CT in Figure 5b-5, respectively.

In this multimodal dataset, we randomly selected a maximum of 1000 images for each class. In this way, a dataset of 45,464 images was selected for 50 different classes in our experiments as shown in Figure 5. Figure 6 shows the examples of experimental data of Figure 5 according to the anatomical district (row) and imaging modality (column). The challenges in our data were high intra-class variance and high inter-class similarity caused by using multiple classes with multiple imaging modalities as shown in Figure 7 and Figure 8. We performed two-fold cross-validation by randomly dividing the whole dataset into almost 50% training and almost 50% testing. In other words, we used half images of C1, C2, C3,…C50 as training and the remaining half of C1, C2, C3,…C50 as testing in the closed-world configuration. In case of open-world configuration, we used all the images of C1, C2, C3,…C25 as training and those of C26, C27, C28,…C50 as testing. In the closed-world configuration, the training dataset mostly contained 500 images per class but in few classes, the number of training images was less than 500, which results in the class imbalance problem [59]. To avoid this problem, we generated some images by data augmentation using image translation and cropping, and in-plane rotation. This data augmentation was performed for only the training dataset.

A similar data augmentation procedure was adopted in the open-world and mixed-world configurations for the training dataset. A detailed description of the training and testing dataset in two-fold cross-validation is given in Table 3, and class imbalance details with augmented images are shown in Table 4.

All the images from each class were resized to 224×224 and converted into a standard bitmap (BMP) file format due to the different size and format of the collected dataset. We used the class label provided in the original datasets for supervised learning. Example images from the selected classes including actual class labels are shown in Figure 5.

Figure 7 and Figure 8 show the degree of intra-class variance and inter-class similarity in our collected dataset, respectively. A significant intra-class variation occurs among different images of a single class as shown in Figure 7. In addition, high inter-class similarity can be observed among different classes as shown in Figure 8. For example, in Figure 8b (cervix MRI) and Figure 8f (bladder MRI), a significant structural correlation can be observed between these two classes. This high degree of intra-class variance and inter-class similarity helps to analyze the performance of different models in a challenging scenario.

In our research study, all the experiments were performed by using a desktop computer with the following specifications: 3.50 GHz Intel^®^ (Santa Clara, CA, USA) Core™ i7-3770K CPU [60] with 12 GB RAM, and NVIDIA (Santa Clara, CA, USA) GeForce GTX 1070 graphics card [61]. This graphics card provides parallel processing capability for both training and testing phase. All the training and testing CNN algorithms are implemented by MATLAB R2018b (MathWorks, Inc., Natick, MA, USA) [62] on the Windows 10 operating system.

### 5.2. Training of CNN Model

Before starting the training process, all the images in the dataset were resized to 224×224×3. In case of images having a single channel such as CT, MRI, X-ray and ultrasound, we made the 3-channel image by copying the image of the 1st channel into those in the 2nd and 3rd channels. The same procedure of copying was performed in case of testing, also.

Our deep residual CNN was then trained by using the stochastic gradient descent (SGD) algorithm [63]. The SGD is the most commonly used algorithm for optimal training of CNNs, and it is very efficient in learning of discriminative linear classifiers with a convex loss function. Its main purpose is to optimize model learnable parameters such as filter weights and biases by taking the derivative of the loss function. In the training process, the correctly labeled data samples are used for the extraction of optimal features. These labeled training data samples passed through the feed-forward stage in the CNN, and then the loss between each actual and desired label is calculated. If the loss value was still greater than a certain threshold, the SGD further optimized the loss function by updating the parameters. The SGD method split the training dataset into mini-batches, performed an iteration for each mini-batch, and then proceeded to learn by defining the time taken for all iterations to complete as one epoch.

The hyperparameters selected for the SGD method in this study were as follows: mini-batch size = 10, learning rate = 0.001, learning rate drop factor = 0.1, learning rate drop period = 10, L2 regularization = 0.0001, and momentum = 0.9. The detailed explanation of each parameter can be found in [64]. During the training process, training data samples were shuffled, and the learning rate was multiplied by the learning rate drop factor for each 10-epoch period. The initial weights used in the FC layer were randomly initialized by using a Gaussian distribution with zero mean and 0.001 standard deviation, and the biases were initialized to zero.

**Figure 9:**
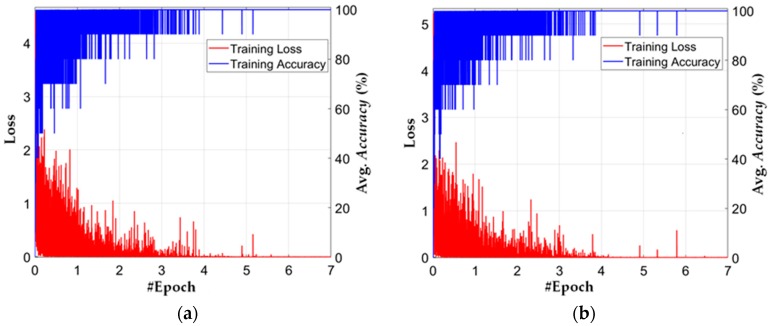
Plot for training loss and accuracy: (**a**) 1-fold cross-validation; (**b**) 2-fold cross-validation.

Figure 9 shows the training loss and accuracy for each epoch from both folds of cross-validations. In all configurations, the loss approaches approximately zero while the training accuracy approaches 100% after a certain number of training epochs, which shows that our deep residual CNN is sufficiently trained with training data. In addition, after performing a number of training experiments for different CNN models, the conclusion is that the fine-tuning of our model results in faster convergence than with conventional training from scratch.

There existed only training and testing data in our experiments, and there was no validation dataset. The best CNN model was selected as follows. As shown in Figure 9, during the total epochs, all the weights of CNN model (whose training loss and accuracy were respectively lower and higher than those of previous model) were updated and stored at each iteration of training. Then, the weights of model which showed the minimum training loss and maximum training accuracy was finally selected as our CNN model.

**Figure 10:**
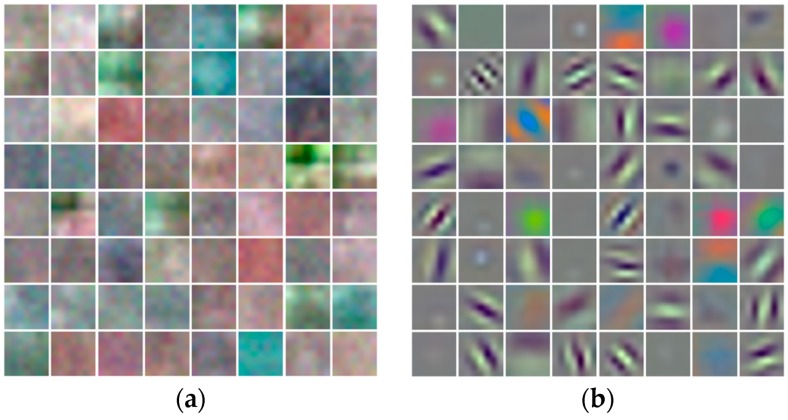
Visualization of learned filters from the first convolutional layer in the case of (**a**) training from scratch with random initialization, and (**b**) fine-tuning with transfer learning.

Figure 10a,b visualize the significant differences in the learned filters from the first convolutional layer Conv1 of Table 2 after training from scratch and fine-tuning, respectively. The learned filters in Figure 10b after fine-tuning were more distinctive as compared to those extracted after training from scratch, as shown in Figure 10a, which shows that more useful features for classification can be extracted by fine-tuning.

### 5.3. Testing and Performance Analysis

The performance of the proposed method was evaluated in term of the average accuracy, average F1.score, mean average prevision (mAP), and mean average recall (mAR) [65], which were calculated as:

(1) $$\mathrm{Accuracy}=\frac{1}{K}\sum_{k=1}^{K}\frac{{\mathrm{TP}}_{k}+{\mathrm{TN}}_{k}}{{\mathrm{TP}}_{k}+{\mathrm{TN}}_{k}+{\mathrm{FP}}_{k}+{\mathrm{FN}}_{k}}$$

(2) $$F1.\mathrm{score}=2\times \frac{\mathrm{mAP}\times \mathrm{mAR}}{\mathrm{mAP}+\mathrm{mAR}}$$

(3) $$\mathrm{mAP}=\frac{1}{K}\sum_{k=1}^{K}\frac{{\mathrm{TP}}_{k}}{{\mathrm{TP}}_{k}+{\mathrm{FP}}_{k}}$$

(4) $$\mathrm{mAR}=\frac{1}{K}\sum_{k=1}^{K}\frac{{\mathrm{TP}}_{k}}{{\mathrm{TP}}_{k}+{\mathrm{TN}}_{k}}\;,$$

where ${\mathrm{TP}}_{k}$ is the true positive, which denotes the correctly classified number of images from class k. ${\mathrm{FP}}_{k}$ is the false positive, which shows the number of images misclassified as class k. ${\mathrm{TN}}_{k}$ is the true negative, which indicates the number of images correctly classified as not belonging to class k. ${\mathrm{FN}}_{k}$ is the false negative, which denotes the number of misclassified images that actually belong to class k. K represents the total number of classes, which equals 50 in our research.

#### 5.3.1. Comparisons of Classification Accuracies by Proposed Modified Residual CNN with Various CNN Models

To evaluate the performance of the proposed deep CNN-based framework for medical image classification, a comparison was made with the most recent deep-learning-based medical image classification and retrieval framework [28,66]. In order to make a fair comparison, the performance of this existing framework was evaluated for our selected dataset. Our proposed method showed a significant performance gain in comparison with [28,66] as shown in Table 5. We also compared the performance of our model with the state-of-the-art methods of CNN models [42,66,68,69,70,71]. The main target of these comparisons was to evaluate the impact of the existing state-of-the-art CNN models in the medical domain. Finally, the impact of transfer learning was explored by training the selected CNN models in two different ways. In the first method, all the models were trained from scratch for our selected dataset. The experimental results for different baseline models are shown in Table 5 without using transfer learning. These results confirm that our modified deep residual model showed the highest average accuracy, and all other selected models also showed performance that was comparable with the existing framework [28].

In the second method, the impact of transfer learning was explored by fine-tuning the top three CNN models on the basis of the results of Table 5 and our modified deep residual model. These selected models were already pre-trained by the ImageNet dataset [72]. For transfer learning, the last few convolutions and all the FC layers (30% of the layers of the complete network) were fine-tuned by our selected dataset, and the filter weights for the initial convolutional layers (70% of the layers of the complete network) were optimized by the ImageNet dataset. The results of transfer learning are reported in Table 6. It can be observed that our modified deep residual model outperformed the other models after applying transfer learning in term of average accuracy, F1.score, mAP, and mAR.

**Figure 11:**
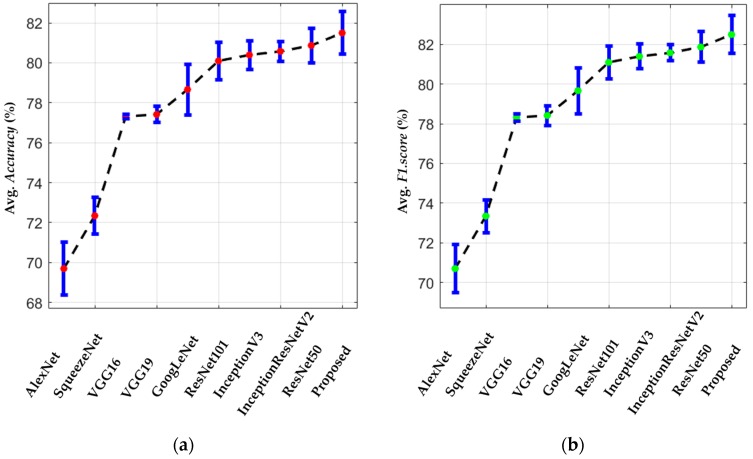
Sensitivity analysis plot of our proposed and various baseline models in terms of (**a**) average accuracy and (**b**) average F1.score (unit: %).

Furthermore, a Monte Carlo simulation setup [73] was created to evaluate the robustness of the various trained CNN models. A detailed analysis is performed in this simulation setup. The performance of each model is iteratively evaluated for a random selection of the testing dataset. In each iteration, 20% of the testing images are selected randomly from the testing dataset, and a total of 20 iterations are performed for both folds. Finally, we calculate the standard deviation and average performance (i.e., accuracy and F1.score) for each model. Figure 11 shows the overall sensitivity performance in term of the average accuracy and F1.score. From the plots, we can see that our modified deep residual model has the best robustness among all the selected models. The second-best model is ResNet50, which also shows comparable performance.

**Figure 12:**
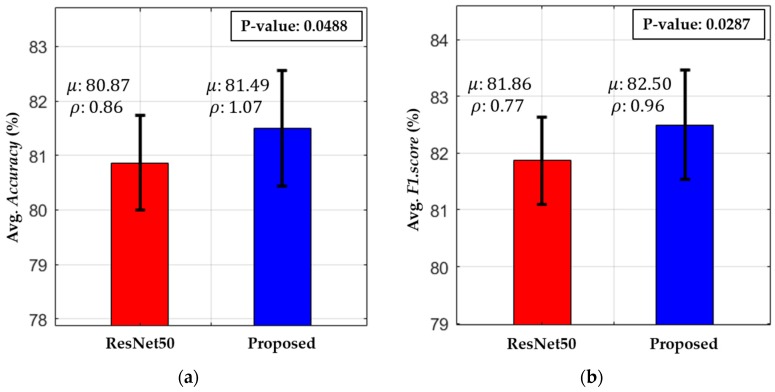
T-test plot of our proposed and the second-best model (ResNet50 [42]) in terms of (**a**) average accuracy and (**b**) average F1-Score (unit: %).

The significance of our modified model was further explored in comparison with the second-best model, ResNet50 [42], by performing a t-test analysis [74]. Figure 12 shows the t-test performance for our modified and the second-best model. The t-test analysis was based on a null hypothesis, in which it was supposed that there was no performance difference between our modified model and the second-best model. After performing a t-test, the experimental results in Figure 12 show that the p-values of accuracy and F1.score for this test were 0.0488 (less than 0.05) and 0.0287 (less than 0.05), respectively. These results show that the null hypothesis for the overall average accuracy was rejected at a 95% confidence level, which indicates that there was a significant difference between the accuracy of our model and that of the second-best model. In addition, the null hypothesis for F1.score was also rejected at a 95% confidence level, which demonstrates the effective performance gain of our modified model in comparison with the second-best model.

**Figure 13:**
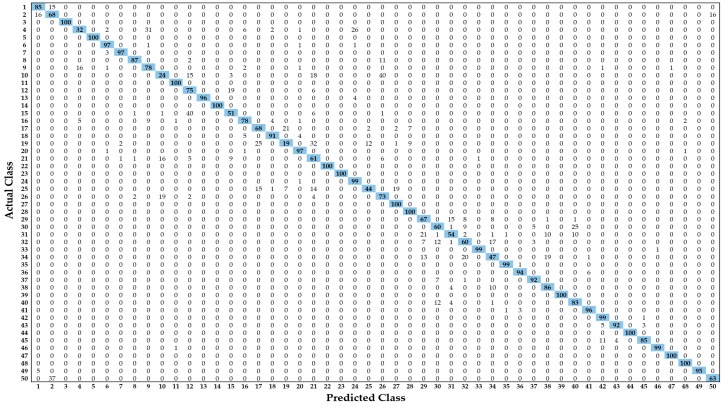
Confusion matrix of the proposed method. The entry in the i-th row and j-th column corresponds to the percentage of samples from class i that were classified as class j.

The more detailed classification performance of our modified deep residual model in terms of the confusion matrix is shown in Figure 13. It can be observed from these results that there were only a few classes that showed a low classification performance, due to the significant structural similarity of the different neighboring body sections. For example, class 10 (i.e., bladder CT in Figure 5a-10) showed low performance because classes 10, 21 (i.e., kidney, renal CT in Figure 5c-1), and 26 (i.e., stomach CT in Figure 5c-6) belonged to neighboring body sections with a significant visual correlation. Similarly, the performance of class 4 (i.e., cervix MRI in Figure 5a-4) was also low due to structure overlapping with class 9 (i.e., bladder MRI in Figure 5a-9) and class 24 (i.e., pancreas MRI in Figure 5c-4). However, the overall performance of our proposed model was good for a heterogeneous dataset with a large number of classes.

#### 5.3.2. Comparisons of Classification Accuracies according to the Features from Different Layers

To investigate whether our modified model can discover the required discriminative features at some intermediate layers, we performed additional experiments. The performance of our proposed and the second-best model (i.e., ResNet50) was analyzed in these experiments. There were two main reasons for comparing our model only with ResNet50 model. The first reason was that both models were similar in layer-wise structure with a small difference (i.e., the difference between the convolutional layer and average pooling layer as explained at the beginning of Section 4.2). The second reason was that all the other models have shown lower performance, which can be seen in the previous performance comparisons in Section 5.3.1.

A total of seven different layers (i.e., Conv2-1, Conv3-1, Conv4-1, Conv5-1, Conv6/AvgPool, FC layer, and Classification layer) were considered for extracting the hidden activation features with the sizes of 802816; 401408; 200704; 100352; 2048; 50; and 50, respectively. These selected features are classified by considering the VNC framework, which we have explained in Figure 4. To make a fair comparison, the same hidden activation features are also extracted from ResNet50. Finally, the average performance is computed for both models (i.e., the proposed model and ResNet50 [42]), which is shown in Table 7. On the basis of the overall performance, we conclude that (1) deeper features are better for the classification task, and (2) the extracted features from the last four layers (i.e., Conv5-1, Conv6, FC layer, and Classification layer) for our modified model are more representative and discriminative in comparison with those from ResNet50.

#### 5.3.3. Comparisons of Classification Accuracies with or without Principal Component Analysis

The discriminative nature of our modified method and the second-best method was further explored by applying principal component analysis (PCA) [75] as a post-processing step. The features of 1×2048 extracted from the last convolutional layer (Conv6 of Table 2) and AvgPool of ResNet50 [42] were projected to the eigenspace separately by applying PCA. A total of 2048 eigenvectors and eigenvalues were obtained. The features obtained after PCA for the testing dataset were classified by using our VNC framework. Various PCA features were selected by considering the different number of eigenvectors for performance analysis.

**Figure 14:**
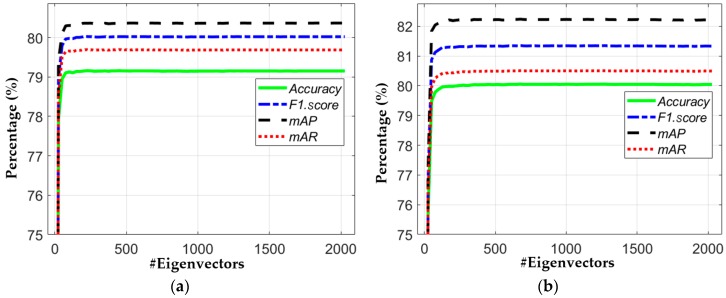
Principal component analysis (PCA)-based performance analysis. (**a**) ResNet50 [42]: features selected from the last average pooling layer; (**b**) proposed model: features selected from the last convolutional layer.

Figure 14 shows the performance of both models according to the number of eigenvectors. On the basis of PCA performance as shown in Table 8, we conclude that the classification performance of PCA was not as good as when using original high-dimension features (extracted from the last convolutional layer Conv6) directly. This shows that the features extracted by our modified model are already diverse. In addition, we can find that the overall PCA performance of our modified method was also high in comparison with ResNet50, as shown in Table 8.

#### 5.3.4. Performance Comparison with Handcrafted Feature-Based Methods

The performance of our modified deep residual method was also compared with that of conventional handcrafted feature-based methods. For a fair comparison, the same dataset was used for all these selected methods. Two known handcrafted feature extraction methods, called LBP [76] and the histogram of oriented gradients (HoG) [77], were considered for feature extraction. Finally, these extracted features were classified by using four different classifiers (i.e., adaptive boosting (AdaBoostM2), multiclass support vector machine (multi-SVM), random forest (RF), and k-nearest neighbor (KNN)) for both feature extraction methods. The classification performance of these selected feature extractions and classification methods is given in Table 9.

It is evident that the proposed deep-CNN-based classification method also outperformed the various handcrafted feature-based methods. There was a significant performance difference between our proposed model (i.e., 81.51%; 82.42%; 83.15%; 81.71%) and the best handcrafted feature-based method (i.e., HoG-KNN shows 70.84%; 70.98%; 71.69%; 70.28%) in terms of classification accuracy, F1.score, mAP, and mAR, respectively.

#### 5.3.5. Closed-World vs. Open-World vs. Mixed-World Configurations

The performance of different classification models (i.e., CNN-based, or handcrafted feature-based) can show a significant performance disparity in different configuration modes. Therefore, it is important to analyze the performance of a model in all possible working scenarios. In this way, the true discriminative nature of a model can be evaluated. Any classification model can be expected to work in the following three configuration modes: closed-world, open-world, and mixed-world. Most of the previous studies [10,28,29,30,31,32,33,34,35,36] have been conducted in a closed-world configuration, which always shows the best performance in comparison to the other modes. The reason for this is that the closed-world configuration is subject to the constraint that all the image categories to be classified in the deployment phase are already known and used in the training phase (the classes in training are same as those used in testing). On the other hand, the open-world configuration is more challenging and is often used in the real environment. In this configuration, the image categories are enrolled during the deployment phase rather than during the training phase, after which classification is performed (the classes in training are different from those in testing). This configuration mode shows the scalable nature of a classification model because the number of image categories can be increased in the deployment phase. Finally, the mixed-world mode includes both open-world and closed-world configurations. In this configuration mode, the categories to be classified in the deployment process may be used during the training phase, and the unseen categories in the training phase can also be enrolled in the deployment phase. In this performance analysis part, the scalable nature of our modified model was explored for all possible configuration modes. Two-fold cross-validation was also performed for both open-world and mixed-world configurations. For each configuration mode, the training and testing dataset was divided in a different way. In the closed-world configuration, the half dataset of C1, C2, C3,…,C50 was used in training and the remaining half in testing. On the other hand, in the open-world configuration, the testing dataset was unseen in the training phase, and thus the dataset was divided in 50% training as C1, C2, C3,…,C25 and in 50% testing as C26, C27, C28,…,C50. Finally, in the mixed-world configuration, the 50% dataset of C1, C2, C3,…,C40 was used in training, and the remaining dataset such as half of C1, C2, C3,…,C40 (i.e., similar to closed-world splitting) and full C41, C42, C43,…,C50 (i.e., similar to open-world splitting) was used in testing.

Table 10 presents the experimental results of our modified model and the second-best method (i.e., ResNet50 [42]) for all these configuration modes. The closed-world and mixed-world configuration results in Table 10 reveal that our modified model outperforms ResNet50. It should also be noted that our model showed the best performance in the open-world configuration, which was more challenging than the mixed-world and closed-world configuration modes. On the other hand, the performance of ResNet50 in the open-world configuration is lower than that in the other configuration modes, which shows the low performance of ResNet50 in real-world situations.

## 6. Discussion

**Figure 15:**
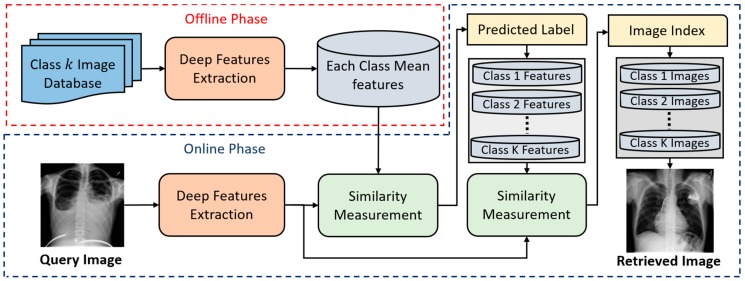
The class-prediction-based content-based medical image retrieval (CBMIR) system by using our proposed deep CNN model.

In general, the efficient image classification is the key part of any CBIR system. In recent few years, the deep learning-based algorithms have shown significant performance gain in image classification tasks. In this proposed work, our main goal is to utilize the strength of deep learning in medical image classification for the CBMIR system. For this purpose, we analyze the performance of different state-of-the-art deep learning models in medical image classification task. In this way, we proposed an enhanced version of existing deep learning model (i.e., ResNet50) which shows the best classification performance in comparison with other models. Finally, based on our enhanced deep learning model, a class-prediction-based CBMIR system is proposed for medical image retrieval as shown in Figure 15.

In this proposed class-prediction-based CBMIR, image retrieval is performed based on class prediction rather than exploring the whole dataset without class prediction. In our proposed class prediction-based retrieval, the key step is to predict the actual class label for the given query image by measuring the similarity score of query image feature vector with the class mean features. In this way, a class label is predicted for the given query image by using the similarity score. Finally, the image retrieval is done by exploring the desired image in predicted class as shown in Figure 15. On the other hand, in without class-prediction-based retrieval, the whole dataset is being explored for a given input query image which is more time taking. A performance comparison is made for both retrieval methods (i.e., with class prediction and without class prediction) by using our modified model and the second-best model (i.e., ResNet50 [42]). It can be observed from Table 11 that class-prediction-based retrieval for our method shows better performance.

It should be noted that our class-prediction-based retrieval method significantly reduces the retrieval time also. The total retrieval time for both methods can be calculated as:

(5) $$\tau_{\mathrm{with}\;\mathrm{class}\;\mathrm{pred}}=\tau_{f.e}+\tau_{f.c}\left(K+n\right)$$

(6) $$\tau_{\mathrm{without}\;\mathrm{class}\;\mathrm{pred}}=\tau_{f.e}+\tau_{f.c}\left(\mathrm{Kn}\right),$$

where $\tau_{\mathrm{with}\;\mathrm{class}\;\mathrm{pred}}$ and $\tau_{\mathrm{without}\;\mathrm{class}\;\mathrm{pred}}$ present the retrieval time for class prediction and without class prediction, respectively. $\tau_{f.e}$ is the feature extraction time for the input query image, and $\tau_{f.c}$ presents the feature comparison time for two feature vectors (i.e., those extracted from the query image and the database image). The entire features database comprises K classes including a total of n feature vectors in each class. From Equations (5) and (6), it can be concluded that the total feature comparison time in the case of class prediction is approximately K times lower than without class-prediction-based retrieval, as K<<n. On the other hand, the total feature extraction time (i.e., $\tau_{f.e}$) remains the same in both cases. To check the validity of Equations (5) and (6) for our proposed class-prediction-based retrieval framework, the total feature extraction and comparison time is measured in both cases. The average feature extraction time $\tau_{f.e}$ for a single query image is obtained as 955 ms in both cases. The total feature comparison times in the case of class prediction and without class prediction are 15.4 ms and 824 ms, respectively. The total feature comparison time in the case of class prediction is approximately 53.5 times lower than that without class-prediction-based retrieval. Finally, we obtain $\tau_{\mathrm{with}\;\mathrm{class}\;\mathrm{pred}}=971\;\mathrm{ms}$ and $\tau_{\mathrm{without}\;\mathrm{class}\;\mathrm{pred}}=1779\;\mathrm{ms},$ which shows that the overall performance of our proposed class-prediction-based retrieval system is much better.

**Figure 16:**
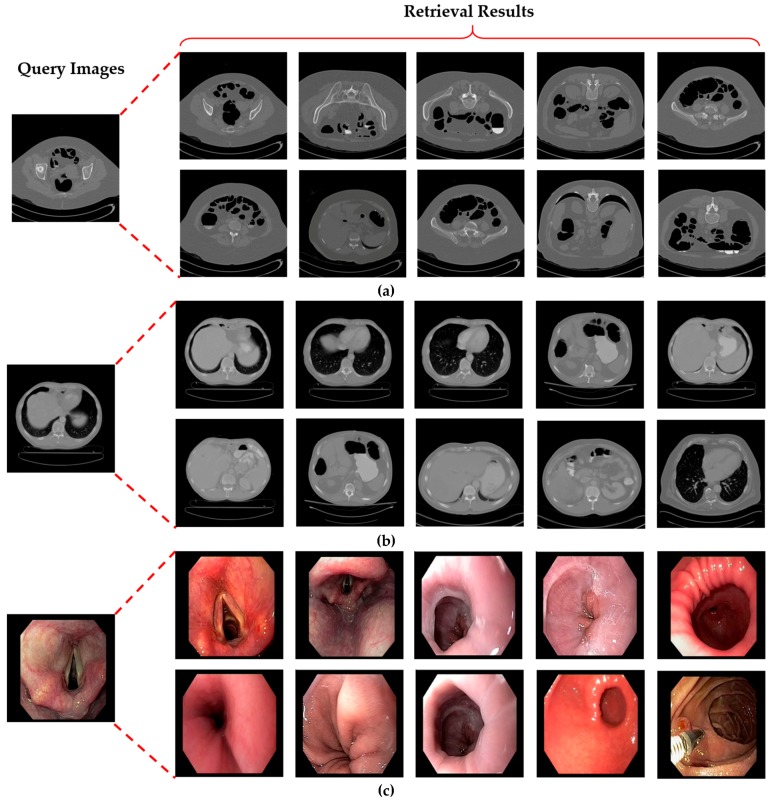

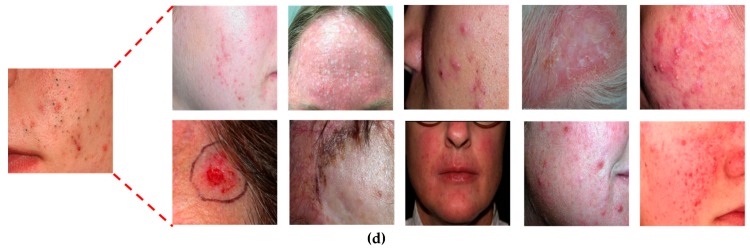
Examples of good retrieval performance of our proposed system for input query image. (**a**) colonography CT; (**b**) liver CT; (**c**) upper endoscopy; (**d**) facial acne.

Figure 16 presents a few examples of correctly retrieved images obtained with our proposed method for different input query images. It can be observed in Figure 16 that the retrieved images have varying illumination, contrast, and high intra-class variance. Despite this challenging nature of the dataset, our method still outperforms with 100% retrieval performance for the selected query images. This shows that our method can be robust to the high intra-class variance of a dataset with the significant performance gain.

**Figure 17:**
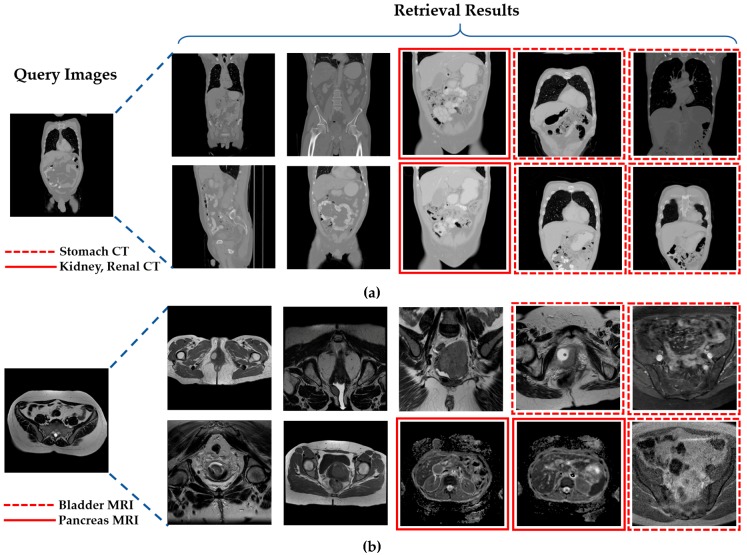
Class-prediction-based false retrieval performance of our proposed system. (**a**) Bladder CT scan as a query image; (**b**) cervix MRI scan as the query image.

A few classes in our collected dataset exhibit low retrieval performance, as shown in Figure 17. The main reason for this performance degradation is the high inter-class similarity among these classes. In Figure 17a, the given query image belongs to the bladder CT scan class, but in the retrieval results, some samples have been misclassified as kidney, renal, and stomach CT scans. This misclassification occurred due to the significant structural overlapping of these three classes, which can also be observed visually in Figure 17. Figure 17b also visualizes the similar structural overlapping of the cervix, bladder, and pancreas MRI scans. However, such misclassification cases can be resolved by adding a feedback mechanism in the proposed retrieval framework. This feedback mechanism will allow the user to explore the given query image in other relevant classes in case of misclassification.

## 7. Conclusions

In this paper, a medical image classification framework is proposed for retrieving heterogeneous medical images by utilizing recent deep learning techniques. The proposed deep-learning-based framework bridges the semantic gap by exploring the discriminative features (i.e., all low-level and high-level features) directly from the images. These extracted features are used to perform class-prediction-based image retrieval tasks. The performance of the proposed system is evaluated on various multimodal databases for all possible real-world configuration modes (i.e., closed-world, open-world, and mixed-world). Our proposed system significantly outperforms the existing retrieval systems used in the medical domain. Moreover, our enhanced ResNet solved the problem of high intra-class variance and inter-class similarity in a medical database, and it improved the classification accuracies. The retrieval performance of the proposed system demonstrates its applicability to various clinical situations, education, and research. Our trained model and image indices of experimental images have been made publicly available to permit other researchers to make performance comparisons. In previous researches on our research topic [28,66], they did not use a validation set for determining the optimal CNN model, but used only training and testing sets like our experiments. In order to maintain fair experimental conditions and comparisons with [28,66] of Table 5, we used only training and testing sets.

In future work, we would compare the accuracies based on the optimal model selection using the additional validation set with our accuracies. We would also study the method which can deal with the case of more classes than 50 classes. In addition, we intended to implement a video-based MIRS for exploring moving sequences. Also, we would further optimize the network by reducing the number of layers and other parameters to make it more efficient.

## Figures and Tables

**Table 1:** Comparison of our proposed and existing methods for medical image classification and retrieval.

Imaging Modalities		Method	Number of Classes	Strength	Weakness
Single modality	CT	Pre-trained CNN [32]	2	Classification performance reaches that of a human observer	Classify only lung cancer CT scan images rather than multiclass images
	X-ray	CNN + edge histogram features are selected [29]	31	High classification performance	Limited dataset (i.e., 1550 images) related to 31 classes (i.e., 50 images in each class) and only 10 images per class are selected for calculating system performance
	CT	Deep CNN model [30]	7	High CAD sensitivity performance with less computation time	Only classify infected and non-infected lung CT scans
	CT	Two-stage multiple instance CNN [33]	12	High classification accuracy	Limited dataset and number of classes
	MRI	3D CNN-based discrimination model [34]	2	High sensitivity	Only classify infected and non-infected brain MRI scans (only 2 classes)
	Fundus camera	CNN + selective sampling (SeS) [35]	2	High average classification accuracy	Uses the reference guide from a single expert
	CT	Restricted Boltzmann machine (RBM) [31]	5	High average classification accuracy	Suitable for smaller representations learning with smaller filters or hidden nodes
	CT	Multiview convolutional network (ConvNets) [36]	2	False positive error is reduced	The CAD sensitivity performance should be enhanced
Multiple modalities	CT, MRI, fundus camera, PET, OPT	Content-based medical image retrieval system (CBMIR) by using CNN [28]	24	High classification accuracy	- A limited number of experimental images - Performance was measured only by closed-world configuration
	CT, MRI, fundus camera, PET, OPT, X-ray, ultrasound, endoscopy, visible light camera	**Proposed method**	50	- High classification performance for multiple modalities data. - Number of classes is much larger than that in the previous work	Using deeper CNN requires more training time

**Table 2:** Layer configuration details of our deep residual convolutional neural network (CNN) architecture.

Layer Name		Feature Map Size	Number of Filters	Kernel Size	Stride	Number of Padding	Number of Iterations
Image input layer		224 × 224 × 3					
Conv1		112 × 112 × 64	64	7 × 7 × 3	2	3	1
Max pool		56 × 56 × 64	1	3 × 3	2	0	1
Conv2	Conv2-1 (1 × 1 Convolutional Mapping)	56 × 56 × 64	64	1 × 1 × 64	1	0	1
		56 × 56 × 64	64	3 × 3 × 64	1	1	
		56 × 56 × 256	256	1 × 1 × 64	1	0	
		56 × 56 × 256	256	1 × 1 × 64	1	0	
	Conv2-2–Conv2-3 (Identity Mapping)	56 × 56 × 64	64	1 × 1 × 256	1	0	2
		56 × 56 × 64	64	3 × 3 × 64	1	1	
		56 × 56 × 256	256	1 × 1 × 64	1	0	
Conv3	Conv3-1 (1 × 1 Convolutional Mapping)	28 × 28 × 128	128	1 × 1 × 256	2	0	1
		28 × 28 × 128	128	3 × 3 × 128	1	1	
		28 × 28 × 512	512	1 × 1 × 128	1	0	
		28 × 28 × 512	512	1 × 1 × 256	2	0	
	Conv3-2–Conv3-4 (Identity Mapping)	28 × 28 × 128	128	1 × 1 × 512	1	0	3
		28 × 28 × 128	128	3 × 3 × 128	1	1	
		28 × 28 × 512	512	1 × 1 × 128	1	0	
Conv4	Conv4-1 (1 × 1 Convolutional Mapping)	14 × 14 × 256	256	1 × 1 × 512	2	0	1
		14 × 14 × 256	256	3 × 3 × 256	1	1	
		14 × 14 × 1024	1024	1 × 1 × 256	1	0	
		14 × 14 × 1024	1024	1 × 1 × 512	2	0	
	Conv4-2–Conv4-6 (Identity Mapping)	14 × 14 × 256	256	1 × 1 × 1024	1	0	5
		14 × 14 × 256	256	3 × 3 × 256	1	1	
		14 × 14 × 1024	1024	1 × 1 × 256	1	0	
Conv5	Conv5-1 (1 × 1 Convolutional Mapping)	7 × 7 × 512	512	1 × 1 × 1024	2	0	1
		7 × 7 × 512	512	3 × 3 × 512	1	1	
		7 × 7 × 2048	2048	1 × 1 × 512	1	0	
		7 × 7 × 2048	2048	1 × 1 × 1024	2	0	
	Conv5-2–Conv5-3 (Identity Mapping)	7 × 7 × 512	512	1 × 1 × 2048	1	0	2
		7 × 7 × 512	512	3 × 3 × 512	1	1	
		7 × 7 × 2048	2048	1 × 1 × 512	1	0	
Conv6		1 × 1 × 2048	2048	7 × 7 × 2048	1	0	1
FC layer		50					1
SoftMax		50					1
Classification layer		50					1

**Table 3:** Summary of training and testing dataset in two-fold cross-validation (unit: images).

Configurations	Validation	Training		Testing	Total
		Original	Augmented		
Closed-world	1st fold	22,732	2268	22,732	47,732
	2nd fold	22,732	2268	22,732	47,732
Open-world	1st fold	21,870	3130	23,594	48,594
	2nd fold	23,594	1406	21,870	46,870
Mixed-world	1st fold	18,435	1565	27,029	47,029
	2nd fold	18,435	1565	27,029	47,029

**Table 4:** Class imbalance details with augmented images for the classes which contain less than 500 images in training (unit: images).

Caption Detail as in Figure 5	Class Name	Class Imbalance Details			
		Original	Augmented	Total	Imbalance Ratio (%)
d-3	Breast mammogram	161	339	500	67.8
e-6	Bones X-rays	169	331	500	66.2
e-9	Shoulder CT	455	45	500	9
e-10	Ankle CT	75	425	500	85
a-1	Hip CT	400	100	500	20
a-2	Knee CT	175	325	500	65
e-2	Facial acne	487	13	500	2.6
e-3	Hand, foot allergies	238	262	500	52.4
e-5	Legs, arms allergies	72	428	500	85.6

**Table 5:** Classification performance of proposed and different baseline CNN models after the training from scratch (unit: %).

CNN Model	Accuracy			F1.score			mAP			mAR		
	Fold1	Fold2	Avg.	Fold1	Fold2	Avg.	Fold1	Fold2	Avg.	Fold1	Fold2	Avg.
AlexNet [28]	71.01	68.41	69.71	71.43	67.84	69.63	72.42	68.21	70.31	70.47	67.47	68.97
SqueezeNet [66]	73.21	71.43	72.32	74.45	73.79	74.12	76.64	75.50	76.07	72.37	72.16	72.27
VGG16 [68]	77.38	77.33	77.36	78.01	78.44	78.22	78.83	79.29	79.06	77.21	77.60	77.41
VGG19 [68]	77.10	77.82	77.46	77.98	78.53	78.25	79.01	79.14	79.08	76.97	77.92	77.45
GoogLeNet [66,69]	79.94	77.37	78.66	80.90	78.11	79.51	82.39	78.08	80.23	79.47	78.15	78.81
ResNet101 [42]	81.08	79.16	80.12	81.81	80.54	81.17	82.85	80.87	81.86	80.79	80.20	80.50
ResNet50 [42]	81.29	79.54	80.42	82.18	80.65	81.41	83.29	80.74	82.01	81.09	80.56	80.83
InceptionV3 [70]	81.17	79.69	80.43	82.24	81.02	81.63	82.98	81.28	82.13	**81.53**	80.76	**81.14**
InceptionResNetV2 [71]	81.11	**80.05**	80.58	82.28	**81.25**	**81.77**	83.46	**81.42**	**82.44**	81.13	**81.09**	81.11
Proposed	**81.84**	79.39	**80.62**	**82.84**	80.16	81.50	**84.41**	80.07	82.24	81.33	80.25	80.79

**Table 6:** Classification performance of proposed and different baseline CNN models in the case of transfer learning (unit: %).

CNN Model	Accuracy			F1.score			mAP			mAR		
	Fold1	Fold2	Avg.	Fold1	Fold2	Avg.	Fold1	Fold2	Avg.	Fold1	Fold2	Avg.
InceptionV3 [70]	79.99	79.46	79.72	80.82	80.38	80.60	82.03	80.26	81.14	79.66	80.49	80.07
InceptionResNetV2 [71]	80.45	78.73	79.59	82.06	79.77	80.92	82.88	79.67	81.28	81.25	79.88	80.56
ResNet50 [42]	82.48	79.33	80.90	83.18	80.62	81.90	84.14	80.90	82.52	**82.24**	80.33	81.28
Proposed	**82.60**	**80.42**	**81.51**	**83.60**	**81.24**	**82.42**	**85.10**	**81.20**	**83.15**	82.15	**81.27**	**81.71**

**Table 7:** Performance comparison of our proposed and the second-best model (Resnet50 [42]) on the basis of feature extraction from different layers (unit: %) (* in our modified layer, average pooling (AvgPool) is replaced by Conv6).

Layer Name	Feature Dim.	ResNet50 [42]				Proposed			
		Accuracy	F1.score	mAP	mAR	Accuracy	F1.score	mAP	mAR
Conv2-1	802816	57.92	58.94	59.39	58.52	57.92	58.95	59.40	58.52
Conv3-1	401408	61.06	62.54	63.25	61.89	61.07	62.55	63.26	61.90
Conv4-1	200704	68.70	69.98	70.61	69.36	68.69	69.97	70.60	69.36
Conv5-1	100352	73.37	75.16	76.06	74.28	74.28	76.27	77.52	75.07
AvgPool/* Conv6	2048	79.25	80.26	80.76	79.77	79.89	81.30	82.31	80.34
FC layer	50	80.17	81.21	81.80	80.63	81.26	**82.43**	**83.33**	81.55
Classification layer	50	**80.90**	**81.90**	**82.52**	**81.28**	**81.51**	82.42	83.15	**81.71**

**Table 8:** Principal component analysis (PCA) performance comparisons of our proposed and the second-best CNN model (ResNet50 [42]) (unit: %).

Option	ResNet50 (No. of Eigenvectors = 170) [42]				Proposed (No. of Eigenvectors = 160)			
	Accuracy	F1.score	mAP	mAR	Accuracy	F1.score	mAP	mAR
With PCA	79.14	79.92	80.14	79.71	80.01	81.32	82.24	80.45
Without PCA	**80.90**	**81.90**	**82.52**	**81.28**	**81.51**	**82.42**	**83.15**	**81.71**

**Table 9:** Comparison of classification performance of the proposed method with different handcrafted feature-based methods (unit: %).

Method	Classifier	Accuracy	F1.score	mAP	mAR
LBP [76]	AdaBoostM2	35.94	35.97	36.02	35.91
	Multi-SVM	45.62	45.48	45.38	45.58
	RF	61.36	61.28	61.52	61.05
	KNN	59.71	59.31	59.39	59.24
HOG [77]	AdaBoostM2	41.37	41.25	41.94	40.58
	Multi-SVM	65.66	67.47	69.51	65.55
	RF	69.54	70.06	71.32	68.86
	KNN	70.84	70.98	71.69	70.28
Proposed		**81.51**	**82.42**	**83.15**	**81.71**

**Table 10:** Closed-world, open-world, and mixed-world performance comparisons of our modified model and ResNet50 [42] (unit: %).

Configuration Mode	ResNet50 [42]				Proposed			
	Accuracy	F1.score	mAP	mAR	Accuracy	F1.score	mAP	mAR
Closed-World	**80.90**	**81.90**	**82.52**	**81.28**	81.51	82.42	83.15	81.71
Open-World	78.56	78.95	79.33	78.56	**82.98**	**83.31**	**83.63**	**82.98**
Mixed-World	79.55	79.49	79.91	79.08	81.33	81.35	81.87	80.84

**Table 11:** Retrieval performance of our proposed model and the second-best model (i.e., ResNet50 [42]) for both methods (i.e., with class and without class prediction) (unit: %).

CNN Model	Without Class Prediction				With Class Prediction			
	Accuracy	F1.score	mAP	mAR	Accuracy	F1.score	mAP	mAR
ResNet50 [42]	80.46	81.58	82.31	80.86	80.90	81.90	82.52	81.28
Proposed	**80.90**	**81.87**	**82.60**	**81.17**	**81.51**	**82.42**	**83.15**	**81.71**
